# Inflamed Pathways to Motherhood: Evaluating Obstetric and Neonatal Outcomes in Rheumatic Pregnancies

**DOI:** 10.3390/jcm14165692

**Published:** 2025-08-12

**Authors:** Batuhan Turgay, Uğurcan Zorlu, Bulut Varlı, Gülşah Aynaoğlu Yıldız, Şahin Kaan Baydemir, Cem Somer Atabekoğlu, Tahsin Murat Turgay

**Affiliations:** 1 Department of Obstetrics and Gynecology, School of Medicine, Ankara University, Ankara 06620, Türkiye; bulutvarli@gmail.com (B.V.); gulsahayna@gmail.com (G.A.Y.); drkaanbaydemir@gmail.com (Ş.K.B.); csatabek@yahoo.com (C.S.A.); 2 Department of Perinatology, Ankara Bilkent City Hospital, Ankara 06800, Türkiye; ugurcanzorlu@gmail.com; 3 Department of Rheumatology, School of Medicine, Ankara University, Ankara 06230, Türkiye; tmturgay@hotmail.com

**Keywords:** rheumatic diseases, pregnancy complications, inflammatory biomarkers, preterm birth, fetal growth restriction

## Abstract

**Objective:** This study aims to evaluate obstetric and neonatal outcomes in pregnancies complicated by RDs and to identify hemogram-derived biomarkers associated with adverse perinatal events. **Methods:** This retrospective cohort study analyzed 360 pregnancies in individuals diagnosed with rheumatoid arthritis (RA), systemic lupus erythematosus (SLE), systemic sclerosis (SSc), ankylosing spondylitis (AS), Sjögren’s disease, sarcoidosis, undifferentiated connective tissue disease (UCTD), and other autoimmune conditions, followed up at the Department of Obstetrics and Gynecology, Ankara University Faculty of Medicine, between 2013 and 2018. Data on disease activity, maternal complications, neonatal outcomes, and inflammatory markers were extracted from electronic medical records. **Results:** Patients with SSc had the highest rates of preterm birth (57.1%) and fetal growth restriction (FGR) (42.9%), whereas those with SLE (50%) and AS (25%) exhibited the highest disease flare rates. Neonates born to mothers with SSc, SLE, and Sjögren’s disease had significantly lower Apgar scores, suggesting increased neonatal distress. NICU admission was associated with elevated neutrophil-to-lymphocyte ratio (NLR) and eosinophil-to-lymphocyte ratio (ELR), with higher NLR and ELR also predicting spontaneous abortion. Monocyte-to-lymphocyte ratio (MLR) and ELR demonstrated the highest predictive value for composite adverse perinatal outcomes. Additionally, RA patients experiencing disease flares had an 87.5% cesarean section (CS) rate, significantly exceeding the general population rate. **Conclusions:** This study underscores the increased risk of preterm birth, FGR, and neonatal complications in RD pregnancies, particularly in SSc and SLE patients. The findings suggest that early risk assessment using hemogram-based inflammatory markers may improve perinatal management and patient stratification.

## 1. Introduction

Rheumatic diseases (RDs) pose a significant challenge in the context of pregnancy due to their systemic inflammatory nature and potential impact on maternal and fetal health. These conditions, including rheumatoid arthritis (RA), systemic lupus erythematosus (SLE), and systemic sclerosis (SSc), can influence pregnancy outcomes by increasing the risk of adverse events such as preterm birth, fetal growth restriction (FGR), and neonatal complications [1,2]. The physiological and immunological changes associated with pregnancy often interact with the underlying disease mechanisms, leading to variable disease activity and pregnancy-related complications [3].

Recent studies have highlighted the importance of multidisciplinary care in managing pregnancies affected by rheumatic diseases. A comprehensive approach involving rheumatologists, obstetricians, and neonatologists is crucial for optimizing maternal and fetal outcomes [4]. Advances in disease-modifying therapies and early risk assessment strategies have improved pregnancy prognosis; however, there remains a need for further research to refine clinical guidelines and individualized treatment plans [5].

A significant concern in rheumatic pregnancies is the increased likelihood of adverse perinatal outcomes, such as neonatal intensive care unit (NICU) admissions and low Apgar scores, particularly in pregnancies complicated by active disease flares [6]. Systemic inflammation and vascular dysfunction contribute to placental insufficiency, which can lead to fetal hypoxia and impaired fetal growth [7]. Moreover, the use of immunosuppressive and biologic therapies during pregnancy requires careful monitoring to balance disease control with potential risks to fetal development [8].

This study aims to evaluate pregnancy and neonatal outcomes in patients with rheumatic diseases by analyzing obstetric complications, neonatal health parameters, and predictive markers of adverse perinatal events. By leveraging a retrospective cohort approach, we seek to provide an insight into the impact of disease activity on pregnancy and highlight potential biomarkers for early risk stratification.

## 2. Material and Methods

This study is a retrospective cohort analysis conducted at the Department of Obstetrics and Gynecology, in the university-based hospital. The study population comprises pregnant individuals diagnosed with RDs who were followed for pregnancy-related outcomes between 2018 and 2023. The patient records were retrospectively reviewed in 2025 to assess the impact of rheumatic diseases on maternal and neonatal health. The inclusion criteria consisted of patients with a confirmed RD diagnosis by the rheumatology clinic, who were monitored and delivered at the Department of Obstetrics and Gynecology during the study period. Only singleton pregnancies were included, while multiple pregnancies were excluded to maintain homogeneity in outcomes. The study focused on patients with RA, ankylosing spondylitis (AS), SLE, Sjögren’s disease, SSc, sarcoidosis, undifferentiated connective tissue diseases (UCTD), and other autoimmune rheumatic conditions.

Patient data were obtained from electronic medical records (EMRs) and hospital archives, and several maternal and neonatal variables were recorded. Maternal characteristics included the presence of rheumatic disease during pregnancy, disease activity and flare-ups, and the development of additional autoimmune conditions. Hemogram parameters, such as the neutrophil-to-lymphocyte ratio (NLR), platelet-to-lymphocyte ratio (PLR), and monocyte-to-lymphocyte ratio (MLR), were also analyzed to evaluate their predictive value for adverse pregnancy outcomes. There are publications indicating that the elevation of these blood parameters may be associated with the activation and flare of relevant rheumatological diseases. Obstetric outcomes included preterm birth (birth before 37 weeks), fetal growth restriction (FGR) (estimated fetal weight is <10%), spontaneous abortion, and composite adverse perinatal outcomes. Neonatal outcomes were assessed through Apgar scores at 1 and 5 min, neonatal intensive care unit (NICU) admission, and neonatal mortality within the first year. For rheumatoid arthritis, a DAS28 score above 5.1 or an SDAI score greater than 26 was accepted as indicating high disease activity or flare. In systemic lupus erythematosus, a SLEDAI-2K score of 10 or higher was accepted as flare. For systemic sclerosis, a modified Rodnan skin score (mRSS) was used, and values exceeding 20 were associated with extensive skin involvement and worse prognosis.

All statistical analyses were performed using SPSS (Version 25, IBM Corp., Armonk, NY, USA). Descriptive statistics were used to summarize the data, presenting continuous variables as mean ± standard deviation (SD) and categorical variables as percentages. Comparative analyses were conducted using independent *t*-tests for continuous variables and chi-square (χ^2^) tests for categorical variables. Logistic regression models were employed to determine significant predictors of adverse pregnancy outcomes, and a *p*-value < 0.05 was considered statistically significant. No sample size was calculated, since all patients whose records were available in the relevant years and who gave birth in our hospital were included in the study.

This study was conducted following the ethical principles of the Declaration of Helsinki, and ethical approval was obtained from the university’s institutional review board (approval number: I25-32234-4, date: 10 January 2025). Due to the retrospective nature of the study, patient consent was waived, and all data were anonymized to ensure confidentiality.

## 3. Results

The study evaluated pregnancy and neonatal outcomes in a cohort of patients diagnosed with RDs, and the final cohort included patients with RA, AS, SLE, SSc, Sjögren’s disease, sarcoidosis, UCTD, and other autoimmune rheumatic conditions. A total of 360 pregnancies were analyzed, focusing on disease activity, obstetric complications, and neonatal outcomes.

### 3.1. Pregnancy and Fetal Outcomes

Among the study population, the presence of rheumatic disease during pregnancy varied across different conditions, with RA (18.2%) and SSc (42.9%) showing moderate prevalence, while SLE (100%) and other autoimmune diseases (100%) were consistently present during pregnancy. Disease flare-ups were most frequent in SLE (50%) and AS (25%), highlighting the challenge of managing active autoimmune conditions during gestation. The highest rates of FGR were observed in SSc (42.9%) and sarcoidosis (50%), suggesting an association between systemic inflammation and impaired fetal development. Preterm birth was significantly more common in SSc (57.1%), underscoring the risk of adverse perinatal outcomes in patients with severe connective tissue diseases (Table 1). In the SSc group, four patients had a Rodnan skin score above 20. In the Sjogren’s disease group, four patients had positive anti-Ro/SS-A antibodies, but no adverse fetal outcome was observed due to these antibodies. In this cohort, the proportion of patients receiving glucocorticoid therapy varied between 6.2% and 15.1% across disease groups. The highest proportion was observed in the sarcoidosis and systemic lupus erythematosus (SLE) groups (12.5%). Eight patients in the RA and AS groups who had flared disease received biological agents.

When the delivery method of all patients was evaluated, it was observed that 55% of the patients (143 patients) gave birth by cesarean section and 45% (117 patients) had vaginal delivery. Of the 143 patients, 50 had a cesarean section due to a previous cesarean delivery. Among 24 RA patients with active disease during pregnancy, 16 (66.7%) underwent cesarean section (CS), while 8 (33.3%) had a vaginal delivery. Among patients who experienced disease flare-ups, seven out of eight (87.5%) delivered via CS, indicating a higher likelihood of surgical intervention in cases of active disease. Previous cesarean section, labor arrest, fetal distress, and joint immobility were the main indications of a cesarean section birth.

### 3.2. Neonatal Outcomes and Apgar Scores

Apgar scores at 1 and 5 min were used as primary indicators of neonatal health. The mean 1 min Apgar scores were lowest in SSc (6.59), Sjögren’s disease (6.52), and SLE (6.65), suggesting higher neonatal distress at birth. Similarly, 5 min Apgar scores followed the same trend, with SLE (7.52) and SSc (7.55) showing the most compromised outcomes (Table 2). This suggests that infants born to mothers with active or severe rheumatic conditions may experience delayed adaptation to extrauterine life.

### 3.3. Hemogram Parameters and NICU Admission

The relationship between maternal hemogram parameters and NICU admission was analyzed. The NLR and ELR were significantly elevated in neonates requiring NICU admission (NLR: 5.76 ± 4.01 vs. 4.84 ± 2.42, *p* = 0.032; ELR: 0.085 ± 0.06 vs. 0.065 ± 0.066, *p* = 0.002), suggesting that these inflammatory markers may serve as predictors of neonatal complications (Table 3). NICU admission indications were mostly transient tachypnea of the newborn, respiratory distress, meconium aspiration syndrome, maternal/newborn infections, low birth weight, and hypoxic–ischemic encephalopathy, respectively.

### 3.4. Predictors of Spontaneous Abortion

Patients who experienced spontaneous abortion had significantly higher NLR (5.87 ± 4.09 vs. 4.74 ± 2.63, *p* = 0.013) and ELR (0.087 ± 0.06 vs. 0.063 ± 0.06, *p* = 0.017) values compared to those with ongoing pregnancies. PLR was also elevated in patients with abortion (188.6 ± 127.31 vs. 161.01 ± 101.42, *p* = 0.039), indicating an association between inflammatory markers and pregnancy loss (Table 3).

### 3.5. Composite Adverse Perinatal Outcomes and Hemogram Parameters

An analysis of hemogram-based predictive markers for composite adverse perinatal outcomes identified MLR (AUC = 0.71, cut-off = 0.31, sensitivity = 87%, specificity = 57%) and ELR (AUC = 0.71, cut-off = 0.079, sensitivity = 43%, specificity = 88%) as the most reliable indicators of risk These findings emphasize the potential of hemogram-derived biomarkers in early risk stratification for high-risk pregnancies in rheumatic disease populations (Table 4).

## 4. Discussion

This study highlights the significant impact of RD on pregnancy outcomes, particularly in relation to preterm birth, FGR, NICU admission, and maternal disease activity during gestation. Our findings align with the existing literature on the subject, while also presenting unique perspectives on biomarkers and predictive models for adverse pregnancy outcomes in RD patients.

One of the key findings of this study is the high incidence of preterm birth and FGR in SSc patients (57.1% and 42.9%, respectively). This result is in line with a 2024 systematic review, which reported a substantially increased risk of preterm labor and intrauterine complications in patients with vascular involvement due to autoimmune disorders [9]. However, unlike Alrifai et al. (2025), who observed higher stillbirth rates in SSc pregnancies, our study did not identify any cases of stillbirth [10]. This difference may be due to improved prenatal care and tighter maternal disease control in our patient population [10].

Additionally, SLE and AS patients in our study exhibited higher rates of disease flare-ups during pregnancy (50% and 25%, respectively). A 2025 meta-analysis of SLE pregnancies demonstrated that active disease at conception was one of the strongest predictors of preterm birth, miscarriage, and hypertensive complications [11]. Our findings reinforce this, as patients experiencing disease flares were more likely to have NICU admissions and lower Apgar scores. However, while Saegusa et al. (2025) found that SLE patients receiving biologic therapy had significantly lower flare rates, our study did not systematically evaluate the role of biologics in pregnancy [12]. Future research should focus on medication safety and the optimization of treatment strategies to prevent disease exacerbation during gestation.

The impact of disease activity on neonatal outcomes was also evident in our findings. Neonates born to mothers with SLE, SSc, and Sjögren’s disease had lower Apgar scores at 1 and 5 min, suggesting higher neonatal distress. These findings align with the results of Williams et al. (2019), who demonstrated that chronic inflammation in RD pregnancies increases the risk of NICU admission and neonatal respiratory distress syndrome [13]. A 2024 systematic review revealed that FGR, preterm birth and extremely low birth weight (<1000 g) baby related with RD patients [14]. Additionally, our study found that NICU admission rates were significantly associated with elevated inflammatory markers, particularly NLR and ELR. 

The clinical utility of biomarkers in predicting adverse perinatal outcomes was one of the strengths of this study. Our results showed that MLR and ELR had high predictive value for composite adverse outcomes, similar to the findings of Saulescu et al. (2024), who proposed that some inflammatory hematological indices could be incorporated into risk assessment models for high-risk pregnancies [15]. However, our study expands on this by demonstrating a specific link between ELR and miscarriage risk, which had not been thoroughly investigated in the prior research.

The rate of cesarean section (CS) in pregnancies with active RA diseases is significantly higher than in the general population. In this study, 66.7% of RA patients underwent CS, and the rate increased to 87.5% in those experiencing disease flares. This is consistent with previous research indicating that RA pregnancies are associated with a higher CS rate due to increased obstetric complications, such as disease activity, joint pain limiting natural labor, and concerns regarding maternal and fetal health [16].

In comparison, a 2019 study by Strouse et al. found that the overall CS rate in RD pregnancies was approximately 44.5%, which is significantly higher than the general population CS rate of 31.8%; but after the propensity-matched analysis they revealed the same CS rate between the groups [17]. Similarly, a 2023 study by Sim et al. demonstrated that RA patients had an adjusted odds ratio of 1.55 for CS, reinforcing that autoimmune inflammation contributes to higher surgical delivery rates [18]. These findings suggest that rheumatic disease activity directly impacts delivery outcomes, supporting our observation that RA flares are associated with a substantially increased likelihood of CS.

Furthermore, a meta-analysis by Lv et al. (2023) reported that women with the high disease activity of RA, were twice as likely to undergo elective or emergency CS due to concerns over fetal distress, preterm labor, and maternal complications [19]. This trend underscores the need for individualized birth planning, considering disease activity, medication use, and overall maternal well-being. In the current study, 43% of the patients had a primary cesarean section, which is a higher rate than normal.

Our study reinforces the findings in the existing literature by demonstrating that RA pregnancies with active disease exhibit an increased CS rate compared to both inactive RA cases and the general obstetric population. These findings highlight the importance of preconception counseling, disease control optimization, and multidisciplinary obstetric–rheumatologic management to improve delivery outcomes in this high-risk group.

From a clinical perspective, these findings emphasize the need for routine disease monitoring and multidisciplinary care in RD pregnancies. Our results suggest that early risk stratification using inflammatory markers may improve pregnancy outcomes, allowing for timely intervention strategies. The high rates of preterm birth and FGR in SSc and SLE patients indicate the necessity of closer fetal surveillance, adjusted medication plans, and early corticosteroid therapy when indicated.

This study’s strengths include its real-world retrospective cohort design, the inclusion of multiple RD subtypes, and the comprehensive evaluation of pregnancy biomarkers. These factors enhance the clinical applicability of our findings, particularly in guiding risk assessment and early intervention strategies.

Despite its strengths, this study has some limitations. The retrospective nature of the data collection may introduce selection bias, the sample size for some disease subgroups (such as SLE and sarcoidosis) was relatively small, and the evaluation of different diseases may be a limitation. Additionally, we did not evaluate the impact of specific immunosuppressive therapies like glucocorticoids and biological agents, which could influence pregnancy outcomes. The reason we did not evaluate this situation is that patients received treatment with very variable protocols and doses. Future prospective multicenter studies with larger sample sizes and standardized disease activity scoring are needed to further validate these findings.

## 5. Conclusions

In conclusion, our study highlights the increased risks of preterm birth, FGR, and neonatal complications in RD pregnancies, particularly in SSc and SLE patients. The association between inflammatory biomarkers and adverse outcomes suggests that early risk assessment using hematological indices may improve perinatal management strategies. Given the growing prevalence of autoimmune diseases among women of reproductive age, further research is needed to develop personalized, biomarker-driven approaches for improving maternal and fetal health in RD pregnancies.

## Figures and Tables

**Table 1:** Pregnancy and fetal outcomes in rheumatologic diseases during pregnancy.

Disease Group	Number of Patients	Presence of Disease During Pregnancy	Disease Flare During Pregnancy	Development of Another Disease During Pregnancy	Presence of Fetal Anomaly	Development of FGR During Pregnancy	Neonatal Death Within First Year	Presence of Preterm Birth
Rheumatoid Arthritis (RA)	132	24 (18.2%)	8 (6.1%)	24 (18.2%)	8 (6.1%)	28 (21.2%)	12 (9.1%)	32 (24.2%)
Ankylosing Spondylitis (AS)	32	16 (50.0%)	8 (25.0%)	12 (37.5%)	0 (0.0%)	12 (37.5%)	0 (0.0%)	8 (25.0%)
Systemic Sclerosis (SSc)	28	12 (42.9%)	4 (14.3%)	8 (28.6%)	4 (14.3%)	12 (42.9%)	0 (0.0%)	16 (57.1%)
Sjögren’s Disease	16	8 (50.0%)	0 (0.0%)	4 (25.0%)	4 (25.0%)	4 (25.0%)	4 (25.0%)	8 (50.0%)
Sarcoidosis	8	4 (50.0%)	0 (0.0%)	0 (0.0%)	0 (0.0%)	4 (50.0%)	0 (0.0%)	4 (50.0%)
Systemic Lupus Erythematosus (SLE)	8	8 (100.0%)	4 (50.0%)	4 (50.0%)	0 (0.0%)	4 (50.0%)	4 (50.0%)	4 (50.0%)
Other Autoimmune and Rheumatic Diseases	24	24 (100.0%)	8 (33.3%)	8 (33.3%)	4 (16.7%)	8 (33.3%)	0 (0.0%)	12 (50.0%)
Undifferentiated Connective Tissue Diseases (UCTD)	12	0 (0.0%)	0 (0.0%)	4 (33.3%)	0 (0.0%)	4 (33.3%)	0 (0.0%)	0 (0.0%)
*p*-value		0.08	0.27	0.04	0.43	0.048	0.21	0.5

Abbreviations: FGR, fetal growth restriction.

**Table 2:** Evaluation of Apgar scores by disease groups.

Disease Group	1 min APGAR	5 min APGAR
Rheumatoid Arthritis (RA)	6.96	7.88
Ankylosing Spondylitis (AS)	6.74	7.74
Systemic Sclerosis (SSc)	6.59	7.55
Sjögren’s Disease	6.52	7.54
Sarcoidosis	7.01	7.72
Systemic Lupus Erythematosus (SLE)	6.65	7.52
Other Autoimmune and Rheumatic Diseases	6.69	7.87
Undifferentiated Connective Tissue Diseases (UCTD)	6.71	7.54
*p*-value	0.038	0.041

**Table 3:** Hemogram parameters predicting NICU admission and abortion in patients with rheumatologic disease during pregnancy.

Parameter	NICU Admission (n = 18) Mean ± SD	No NICU Admission (n = 58) Mean ± SD	*p*-Value
Neutrophil-to-Lymphocyte Ratio (NLR)	5.76 ± 4.01	4.84 ± 2.42	0.032
Monocyte-to-Lymphocyte Ratio (MLR)	0.32 ± 0.14	0.28 ± 0.11	0.111
Platelet-to-Lymphocyte Ratio (PLR)	184.91 ± 124.81	164.29 ± 68.23	0.044
Eosinophil-to-Lymphocyte Ratio (ELR)	0.085 ± 0.06	0.065 ± 0.066	0.002
White Blood Cell/Neutrophil Ratio (WBC/Neutrophil)	1.46 ± 0.19	1.51 ± 0.19	0.067
Platelet–Monocyte Ratio (PMR)	638.19 ± 224.64	670.5 ± 280.57	0.029
	**Abortion Present (n = 20) Mean ± SD**	**No Abortion (n = 76) Mean ± SD**	***p*-Value**
Neutrophil-to-Lymphocyte Ratio (NLR)	5.87 ± 4.09	4.74 ± 2.63	0.013
Monocyte-to-Lymphocyte Ratio (MLR)	0.33 ± 0.14	0.27 ± 0.11	0.082
Platelet-to-Lymphocyte Ratio (PLR)	188.6 ± 127.31	161.01 ± 101.42	0.039
Eosinophil-to-Lymphocyte Ratio (ELR)	0.087 ± 0.06	0.063 ± 0.06	0.017
White Blood Cell/Neutrophil Ratio (WBC/Neutrophil)	1.48 ± 0.21	1.49 ± 0.19	0.231
Platelet–Monocyte Ratio (PMR)	650.95 ± 226.12	657.09 ± 274.95	0.154

**Table 4:** Hemogram parameters predicting composite adverse perinatal outcomes.

Parameter	AUC	Cut-Off Value	Sensitivity	Specificity
Neutrophil-to-Lymphocyte Ratio (NLR)	0.66	5.65	0.59	0.77
Monocyte-to-Lymphocyte Ratio (MLR)	0.71	0.31	0.87	0.57
Platelet-to-Lymphocyte Ratio (PLR)	0.65	177.21	0.51	0.87
Eosinophil-to-Lymphocyte Ratio (ELR)	0.71	0.079	0.43	0.88
White Blood Cell/Neutrophil Ratio (WBC/Neutrophil)	0.49	1.49	0.21	0.91
Platelet–Monocyte Ratio (PMR)	0.56	655.48	0.84	0.44

## Data Availability

The original contributions presented in this study are included in the article. Further inquiries can be directed to the corresponding author.
